# Eccentric Exercise and Muscle Damage: An Introductory Guide

**DOI:** 10.3390/jfmk11020139

**Published:** 2026-03-26

**Authors:** Vassilis Paschalis, Nikos V. Margaritelis, Panagiotis N. Chatzinikolaou, Anastasios A. Theodorou, Michalis G. Nikolaidis

**Affiliations:** 1 School of Physical Education and Sport Science, National and Kapodistrian University of Athens, 17237 Athens, Greece; 2 Department of Physical Education and Sport Science at Serres, Aristotle University of Thessaloniki, 62110 Thessaloniki, Greece; nvmargar@auth.gr (N.V.M.); chatzinpn@phed-sr.auth.gr (P.N.C.); nikolaidis@auth.gr (M.G.N.); 3 Department of Life Sciences, School of Sciences, European University Cyprus, Nicosia 2404, Cyprus; a.theodorou@euc.ac.cy

**Keywords:** exercise induced muscle damage, nutrition and recovery, repeated bout effect, skeletal muscle adaptations, unaccustomed exercise

## Abstract

At the dawn of the 20th century, seminal studies revealed that muscle fibers produce less heat and generate greater force during elongation than during shortening actions, laying the foundation for contemporary research on eccentric exercise. Today, eccentric exercise is widely used by athletes to enhance strength and by older adults to maintain functional capacity, yet it may cause muscle damage, particularly in unaccustomed muscles. Despite more than a century of investigation, the precise mechanisms of eccentric exercise-induced muscle damage remain incompletely resolved. Nevertheless, eccentric exercise serves as a valuable model for studying muscle injury and repair and adaptation. This review organizes current evidence into nine key themes: (1) eccentric exercise-induced muscle damage and flawed biomarkers, (2) satellite cell-mediated and alternative repair pathways, (3) high-force, low-cost contractions and metabolic impact, (4) repeated bout effect and protective adaptations, (5) architectural remodeling of fascicles, sarcomeres and tendon, (6) distinct neural control, proprioception, and cross-education adaptations, (7) mitochondrial, sarcoplasmic reticulum, and cytoskeletal stress remodeling, (8) connective tissue perturbation, remodeling, and joint stability, and (9) targeted, cautious use of antioxidant supplementation. Rather than offering a comprehensive overview, this review highlights pivotal experiments, concepts, and controversies within these themes to guide readers to the most impactful discoveries in eccentric exercise and muscle damage.

## 1. Introduction

At the turn of the twentieth century, pioneering observations in the physiology of skeletal muscle contraction were made. It was discovered that muscle fiber elongation during contraction (work performed by a muscle that resists an external force) produces less heat [1,2] and generates greater force output [3] compared to muscle fiber shortening during contraction (work performed by the muscle). Soon after the initial physiological studies, it was discovered that eccentric exercise is associated with muscle damage, leading to the birth of a fruitful area of research: exercise-induced muscle damage. Exercise-induced muscle damage was linked to the earlier recognized condition of delayed onset muscle soreness [4,5].

During this early era of exercise physiology, researchers faced challenges in formulating theories and answering fundamental questions about both the characteristics of muscle contraction and the recovery processes of muscle tissue, particularly in response to interventions involving large eccentric components. While some early theories served their purpose for a time, they have since been surpassed and should now be considered outdated. In contrast, other theories have remained robust and are still widely accepted, thanks to improved methodologies, stronger theoretical foundations, and more refined interpretative frameworks.

In the early stages of exercise physiology, little attention was given to exercise-induced muscle damage. As noted by Hoppeler [6], physiologists at the time tended to view skeletal muscle as a homogenous and uninteresting tissue, at least until Holloszy’s ground-breaking observation in 1967 that endurance training induces mitochondriogenesis [7]. Holloszy’s discovery sparked a resurgence of interest in muscle physiology, reigniting research into exercise-induced muscle damage. Subsequent studies focused on unravelling the mechanisms behind muscle damage at both molecular and physiological levels, significantly advancing our understanding of muscle physiology.

### The Approach of the Present Review

Despite the exponential increase in publications on eccentric exercise-induced muscle damage, relatively few studies have explicitly focused on the underlying mechanisms of this damaging process. Likewise, the present review does not address functional outcomes, practical applications, or training recommendations in detail; instead, it provides a mechanism-driven and historically grounded synthesis of eccentric exercise-induced muscle damage per se. With this in mind, the topics covered in this review have been selected for their potential to unravel the mechanisms driving eccentric exercise-induced muscle damage. More importantly, this review will present not only milestone findings but also revisit pioneering methodologies that have been forgotten or discontinued over time. We propose that these methodologies, when combined with contemporary technology and advanced study designs, could now be revised and applied to current research to address long-standing mechanistic questions.

A critical issue in interpreting the eccentric exercise literature is that “eccentric exercise” does not represent a single standardized stimulus. Studies differ substantially in exercise modality (e.g., downhill running, isokinetic dynamometry, eccentric cycling, stair descent, electrically stimulated contractions), as well as in loading parameters such as intensity, number of contractions, total work, contraction velocity, muscle length, frequency, and intervention duration. These protocol differences are likely to contribute substantially to the variability in the magnitude of muscle damage, inflammatory responses, neuromuscular impairment, and long-term adaptation reported across studies. Therefore, where relevant, the present review also considers the specific characteristics of the eccentric protocols discussed, so that mechanistic findings can be interpreted within the context of the loading conditions under which they were obtained.

This review also incorporates multidisciplinary perspectives, including muscle physiology (e.g., mechanisms of eccentric exercise-induced muscle damage and energy expenditure during eccentric exercise), biomechanics (e.g., architectural and morphological adaptations to eccentric exercise), biochemistry (e.g., redox and anti-inflammatory adaptation processes in response to eccentric training), and nutrition (e.g., nutritional interventions in exercise-induced muscle damage). By bringing together these disciplines within the specific context of eccentric exercise-induced muscle damage, we aim to provide an integrated framework that goes beyond the scope of previous reviews and deepens our understanding of the responses and adaptations to eccentric exercise-induced muscle damage.

Our aim is to address unresolved questions posed by early researchers and highlight areas that remain open for discussion in the research area of eccentric exercise-induced muscle damage, providing directions for future research. To achieve this goal, we will present ten key aspects of eccentric exercise-induced muscle damage that have captured researchers’ attention over the past 130 years. For each characteristic, we will explore the mechanistic evidence, illustrating how these concepts have either endured or evolved over time. To our knowledge, this is the first review to systematically follow these concepts across more than a century of research, juxtaposing early mechanistic hypotheses with contemporary data and methodological advantages. Our objective is not to provide a comprehensive description of exercise physiology related to eccentric muscle function as a whole but to focus on some specific aspects of eccentric exercise that are central to understanding its mechanisms and guiding future experimental work.

## 2. Mechanisms Involved in Eccentric Exercise-Induced Muscle Damage

At the beginning of the 20th century, exercise-induced muscle soreness was first described in vivo by Hough [4,5]. He proposed that untrained muscles engaged in eccentric exercise could experience connective tissue rupture or nerve fiber injury, leading to delayed onset muscle soreness (evidenced by ergographic tracing, which depicts the amplitude and time course of repeated muscle contractions and the progressive decline in force or work output over time) [4,5]. The link between unaccustomed exercise and muscle damage was further emphasized when it was observed that arm muscles are more susceptible to damage compared to leg muscles, which are considered relatively trained due to their use in everyday activities [8].

Early investigations often used tetanic muscle contractions in vitro to simulate eccentric exercise (muscle stretching or lengthening), but this approach led to irreversible stretching of the muscle’s elastic components—an effect considered a potential cause of muscle soreness [3,9]. It was mistakenly suggested that exercise-induced muscle soreness might result from anaerobic metabolism [10]. However, understanding of anaerobic metabolism was still in its early stages, having been described only a few years earlier [11,12,13]. Additionally, it was suggested that during high-intensity eccentric contraction (*muscle lengthening*), different regions of the muscle might behave differently. Specifically, one region might “hold”, allowing the stretch to be reversible, while another region might “give”, resulting in an irreversible stretch [3,14,15].

The limited interest of early researchers in exercise-induced muscle damage can be attributed to the prevailing view of skeletal muscle as an inert tissue [6]. In the early 50s, Hill [16] noted that his own experience of muscle soreness after participating in activities with large eccentric components could not be explained. Around this time, researchers reported for the first time that eccentric exercise (e.g., stepping down with one limb) caused greater muscle soreness than concentric exercise (e.g., stepping up with the other limb) [17].

The greater mechanical stress on muscle fibers or/and the overstretching of muscle’s elastic components during eccentric contractions were proposed as possible mechanisms for this soreness [17,18]. The “spasm theory” (i.e., an involuntary muscular contraction) was also proposed as an explanation for greater soreness following eccentric contractions, based on higher unipolar electromyographic activity (i.e., multiple input electrodes are amplified relative to a single reference) in sore muscles after eccentric exercise [19]. However, this theory was rejected when bipolar, rather than unipolar, electromyography was used [20].

The initial observation of the delayed onset muscle soreness (DOMS) as a consequence of exercise involving large eccentric components led researchers to investigate the underlying causes and mechanisms. To answer questions about the origin of muscle soreness, they focused on the molecular aspects of skeletal muscle. The advent of electron microscopy enabled researchers to describe, for the first time, the morphological disorganization of muscle fibers in response to eccentric exercise. It was revealed that although no ultrastructural disorganization was initially apparent, significant muscle damage became evident two days after downhill running (which involves muscle lengthening contractions). This damage was characterized by broadening, streaming and disruption of the Z-band [21]. It was also found that eccentric exercise had particularly detrimental effects on type II muscle fibers. Initial activation of lysosomal enzymes and the resulting inflammation were followed by disturbances in the Z-band [22,23,24].

These findings proposed that exercise-induced muscle damage may result from the excessive influx of calcium intracellularly [25,26], which activates calcium-dependent proteases, such as calpains [27,28], due to the high mechanical forces involved. Desmin and a-actinin, key components of Z-line and substrates of calpains, undergo degradation after eccentric exercise, directly compromising the integrity of the Z-line [29,30]. Additionally, Yu and colleagues [31] suggested that desmin plays a role in sarcomere repair and remodeling in response to eccentric exercise.

### 2.1. Contemporary Theories About the Mechanisms Involved in Muscle Damage

The initial causes of exercise-induced muscle damage are thought to be both biochemical and physiological. However, the distinction between these two initiators of muscle damage is often unclear, making it difficult to establish a direct cause-and-effect relationship. In the late 1980s, xanthine oxidase was identified as a significant source of reactive oxygen species during exercise [32]. Over a decade later, research showed that elevated xanthine oxidase levels following exhaustive exercise were implicated in exercise-induced muscle damage [33,34]. It was also hypothesized that the oxidation of cysteine residues could activate NF-kB, a key trigger of inflammation and muscle damage [35,36].

Physiologically, muscle damage has been attributed to the heterogeneous lengthening of sarcomeres when they are stretched beyond their optimal length. This can lead to the mechanical instability of the weakest sarcomeres, a concept known as the “popping sarcomere theory” [37,38,39]. Non-uniform lengthening of sarcomeres can cause shearing of myofibrils and expose membranes to large deformations and damage [40]. The “popping” sarcomere theory partially aligns with earlier ideas that different regions of a muscle respond differently to eccentric contraction, leading to irreversible stretching in some areas [3,14,15]. However, it has also been observed that 24 h after voluntary eccentric exercise there was no substantial damage to the Z-line or the myofibers that were analyzed, suggesting that such structural disruption may depend on the specific characteristics and intensity of the eccentric loading [41,42].

The structural non-uniformities within sarcomeres may also explain the phenomenon of residual force enhancement—a higher force output that persists after a muscle fiber has been stretched [43]. This is thought to occur because some sarcomeres, with increased myofilament overlap, compensate for weaker “popped” sarcomeres [44]. The structural giant protein titin, which stabilizes sarcomeres during lengthening, is believed to contribute to this passive force enhancement [45,46], supporting earlier hypotheses that muscle’s elastic components play an active role during lengthening contractions [3,9].

### 2.2. Biomarkers for the Assessment of Muscle Damage

**Figure 1:**
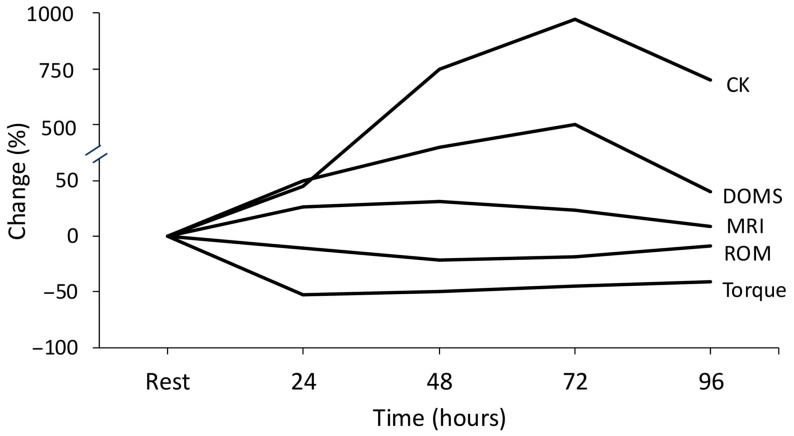
Time course of percent change from pre-exercise values for a direct marker of muscle damage [Magnetic Resonance Imaging (MRI; data from [51])] and common indirect muscle damage biomarkers [Torque; data from [52]; Range of Motion (ROM; data from [53]); Delayed onset muscle soreness (DOMS; data from [53]); Creatine Kinase (CK; data from [54])], following unaccustomed eccentric exercise.

The most reliable indirect biomarkers for the assessment of exercise-induced muscle damage include a decline in strength output, a reduced pain-free range of motion (both of which are often described as muscle stiffness) [25], delayed onset muscle soreness and elevated levels of the enzyme creatine kinase [47] (Figure 1). Furthermore, recent data indicate that limb dominance does not influence the magnitude of exercise-induced muscle damage in the knee extensors of young women [48]. Muscle stiffness results from both fluid accumulation (edema) and calcium release from the sarcoplasmic reticulum of damaged muscle fibers [25]. Each of these biomarkers has distinct physiological mechanisms [49], and early gene expression patterns linked to each marker differ significantly [50]. Therefore, these biomarkers should not be used interchangeably to assess “muscle damage”.

Creatine kinase, one of the more frequently measured proteins following eccentric exercise [55], accumulates in the blood due to increased membrane permeability or muscle fiber membrane breakdown [56,57] (Figure 1). The protocol for measuring creatine kinase activity was introduced in 1954 [58], and a few years later, creatine kinase activity was measured after an intense exercise session [59]. Since then, creatine kinase measurement has been included in nearly every protocol involving unaccustomed exercise or fatigue interventions. However, creatine kinase concentration exhibits substantial inter-individual variability, which makes this biomarker an unreliable indicator of the extent of muscle damage [60]. Similarly, despite the fact that muscle biopsies are considered the gold standard for assessing muscle damage, they have been criticized for potential inaccuracies, as repeated biopsies may contribute to inflammation and DOMS [61,62]. Additionally, extrapolating biopsies data from animal studies to humans is problematic, as animals are typically subjected to very high intensity and volume of electrical stimulation to induce muscle damage [31,63,64].

Understanding exercise-induced muscle damage has significantly evolved since its initial description. While DOMS and creatine kinase remain widely used as indirect biomarkers of muscle damage, they should be interpreted cautiously, as they (i) exhibit considerable variability between individuals [65], (ii) DOMS do not always correlate with serum creatine kinase levels [66], and (iii) both biomarkers do not necessarily correlate with the actual extent of muscle fiber damage [67]. Mechanistic insights, such as the role of sarcomere instability, oxidative stress, and proteolytic enzyme activity, provide a deeper understanding of how muscle damage occurs and how it can be mitigated through training adaptations and recovery strategies.

It has been suggested that the most widely accepted explanation for DOMS is that it results from microdamage to muscle fibers followed by an inflammatory response [68]. However, structural changes typically considered indicative of muscle damage do not consistently match the time course or severity of soreness [49]. This discrepancy prompted a re-evaluation of the relationship between tissue injury and DOMS, with exercise intensity emerging as a key factor for quantitatively linking soreness and damage [68]. In addition, muscle soreness after unaccustomed exercise may result from the action of nerve growth factor (NGF), which is produced by inflammatory cells such as macrophages and mast cells [69] and acts downstream of bradykinin, thereby contributing to the maintenance of mechanical hyperalgesia [70]. NGF has been found to induce hyperalgesia both rapidly, by sensitizing peripheral nociceptors, and later, by altering the expression of neurotransmitters, neuromodulators, and ion channels in dorsal root ganglion neurons [71].

The cause-and-effect relationship between eccentric exercise and muscle damage, first described by early researchers, remains well-established today. Current evidence suggests that the primary cause of muscle damage is not the muscle lengthening *per se*, but rather the unaccustomed high tension experienced during eccentric (lengthening) muscle contraction. Indeed, the greater muscle damage observed during eccentric compared to concentric contractions is attributed to the higher tension generated during the former. Moreover, a previously overlooked finding from our group demonstrated that eccentric exercise performed at shorter muscle length—where higher muscle tension was produced—resulted in greater changes in muscle damage biomarkers than eccentric exercise performed at a longer muscle length with lower muscle tension [72]. Further supporting the role of unfamiliarity in exercise-induced muscle damage, comparable muscle damage was observed between concentric and eccentric exercise when both were performed at long muscle length [73].

Along with muscle length during eccentric contractions, the velocity of movement also appears to play a crucial role in exercise-induced muscle damage. Relevant studies indicate that contraction velocity is an important determinant of the extent of muscle damage induced by eccentric exercise [74,75,76]. Specifically, when the same muscle group is exercised eccentrically at different movement speeds, fast-velocity eccentric contractions, compared to slow-velocity contractions, consistently produce larger decrements in muscle strength and greater increases in delayed onset muscle soreness, even when the overall work performed is comparable [74,75,76]. These findings suggest that higher lengthening velocities amplify the mechanical strain imposed on active fibres, thereby exaggerating the subsequent functional impairment and subjective symptoms of muscle damage. Recent work has also emphasized the need to distinguish between eccentric muscle actions, eccentric training, and eccentric overload, clarifying that muscle damage should not be attributed to “eccentricity” alone, but more specifically to the unaccustomed high mechanical tension and loading conditions that may accompany eccentric exercise [77].

### 2.3. Take Home Message

The study of exercise-induced muscle damage has progressed considerably, yet many misconceptions persist. The notion that soreness equates to muscle growth is misleading, and a more nuanced understanding of the physiological mechanisms underlying muscle damage is necessary. Future research should focus on refining non-invasive biomarkers for muscle damage assessment and exploring how individual variability influences responses to eccentric exercise. Moreover, applying these insights to training methodologies can optimize recovery and enhance performance while minimizing the risk of injury.

## 3. Skeletal Muscle Repair

Early researchers recognized the damaging effects of eccentric (muscle lengthening) exercise and understood that skeletal muscle could fully recover after a few days. However, they lacked the tools to detail the extent of “muscle damage” or describe the physiological repair processes involved.

Distinguishing between degenerative and regenerative processes in muscle tissue repair after eccentric exercise is challenging [78]. Muscle fibers themselves lack intrinsic regenerative ability, relying on muscle stem cells, known as satellite cells, for repair [79]. These satellite cells, first identified in frog muscle [80], were later shown to proliferate and contribute to muscle regeneration after fiber damage. Observations of mitotic structures near damaged muscle cells following exercise reinforced the role of satellite cells in muscle repair [81]. Initially, there was scepticism about the ability of human satellite cells to transform into myoblasts after muscle damage [82], but later research established that satellite cells serve as precursors for muscle regeneration, similar to processes seen in embryonic myogenesis [83].

Satellite cells originate from the dermomyotome during development [84] and are named for their position on the periphery of muscle fibers [80]. Located between the plasma membrane of muscle fibers and the basement membrane, satellite cells maintain structural integrity and regulate muscle cell activity, partly by providing growth factors [85]. Their positioning ensures that satellite cells can respond to signals from both the muscle fiber and the surrounding tissue [86]. Additionally, satellite cell fate is influenced by the distinct receptors present at the two opposing sides—the muscle fiber plasma membrane and the basement membrane. Based on this, it has been suggested that the cell in contact with the muscle fiber differentiates and is incorporated into the fiber as a new myonucleus, whereas the cell in contact with the basement membrane remains a satellite cell, thereby replenishing the satellite cell pool [87]. In their quiescent state, satellite cells remain inactive until muscle injury occurs, at which point they are activated and begin proliferating to facilitate muscle repair [86,88]. They are typically identified by the expression of the transcription factor paired box 7 (Pax7) [89,90].

Early research on satellite cells focused on their anatomical characteristics, such as their location, size, shape, and mitotic capacity [91,92]. Interest in satellite cells grew when animal studies showed that their population decreases with age [93], but increases in response to hypertrophy [94]. A few years later, satellite cell activation in response to eccentric exercise was linked to muscle repair, suggesting that mitogenic factors and growth factors released from the damaged myofiber regulate satellite cell activity [95,96].

Exercise-induced muscle damage disrupts the capillary system, but during muscle regeneration, microvascular networks are reorganized, restoring blood flow regulation [97,98]. This restoration supports myofiber regeneration. Enhanced muscle capillarization has been shown to promote satellite cell activation and speed recovery after eccentric contractions [99]. Additionally, research has indicated that satellite cell proliferation following eccentric exercise coincides with increased markers of ribosome biogenesis, suggesting improved transcription and translation control for muscle adaptation [100].

The transition of satellite cells from a quiescent to an active state is partly facilitated by the accumulation of leucocytes-mediated substances at the injured site. The cyclooxygenase pathway, which promotes inflammation, is central to this process [101]. Macrophages, by releasing cytokines, further support muscle regeneration by enhancing myoblast proliferation [102]. Recent studies suggest macrophages may act similarly to senescent cells, influencing muscle repair by regulating inflammation and supporting satellite cell function [103]. However, the role of macrophages following muscle injury remains debatable, as this state may be just one of many macrophage activation states [104].

During the acute phase of muscle injury, inflammatory cells secrete enzymes to break down damaged tissue [42,105], while reactive oxygen species contribute to muscle degeneration [106,107,108]. However, widespread use of non-steroidal anti-inflammatory drugs (NSAIDs) has been documented for elite speed and power athletes [109]. NSAIDs act by inhibiting the activity of cyclooxygenase which derives from arachidonic acid and is the key enzyme in the synthesis of prostaglandin [110]. It has also been suggested that inhibition of COX activity by ingestion of NSAIDs suppresses the increased mixed muscle protein synthesis rates normally observed following exercise [111]. However, while the precise mechanism by which the COX pathway acts on satellite cells and the ensuing events in the repair or hypertrophy process remains unclear, there is mounting evidence for a positive regulatory effect on satellite cell activity [112]. Specifically, studies show that inhibiting inflammation with NSAIDs attenuates the exercise-induced increase in satellite cells number, which may impair muscle regeneration or hypertrophy [111,112]. However, these findings, mostly from animal studies, should be interpreted with caution [113].

**Figure 2:**
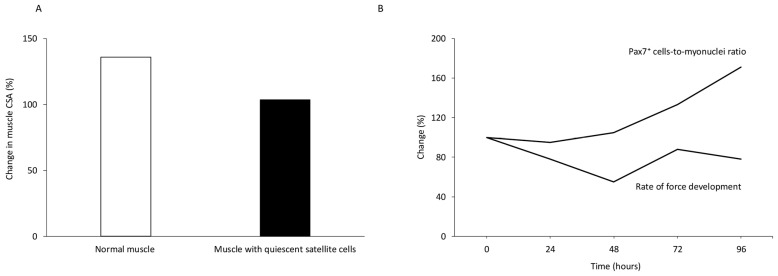
(**A**) Following eccentric exercise, muscles in which satellite cells remain quiescent exhibit attenuated hypertrophy (data from [116]). (**B**) Change in rate of force development (RFD) and the Pax7^+^ cells-to-myonuclei ratio after a single bout of eccentric exercise (data from [41]).

It is well-established that satellite cells are crucial for muscle repair following highly damaging exercise [114,115] (Figure 2A,B). Severe eccentric exercise can lead to significant impairments if satellite cells are absent [116,117,118]. Even a reduced number of satellite cells can negatively affect muscle adaptation, such as decreasing muscle fiber cross-sectional area [119]. Interestingly, satellite cell proliferation can also occur in response to non-damaging exercise [120], possibly serving to preserve the satellite cell pool [113,118].

While satellite cells are crucial for muscle regeneration, skeletal muscle can manage tissue damage from eccentric exercise without always relying on them [89,117]. Milder muscle damage that preserves the regional microvasculature is believed to prompt robust regeneration through other mechanisms [121]. Cell-autonomous membrane repair mechanisms play a key role in muscle recovery after mild damage [122,123,124,125].

In response to eccentric exercise, myofibril repair and sarcomere reorganization are supported by migration of subsarcolemmal nuclei. This helps deliver the mRNA needed for protein synthesis to repair damaged sarcomeres [126]. Annexins also assist in sealing the plasma membrane at injury sites by forming a cap over the injured site [124], and mitochondria localize nearby to regulate calcium removal [126,127].

### Take Home Message

Satellite cells play a fundamental role in muscle repair and regeneration following eccentric exercise, but muscle adaptation can also occur independently of their involvement. Moreover, despite significant advances in understanding satellite cell biology, their role in muscle adaptation beyond injury recovery remains unclear. Moving beyond traditional damage models could lead researchers to explore satellite cell function in aging, exercise-induced adaptation, and non-damaging exercise stimuli [128].

## 4. Energy Expenditure

### 4.1. Energy Expenditure During Eccentric Exercise

Early research explored the energy demands of different muscle contractions. In an initial in vitro study, it was observed that lengthening contractions (eccentric) produced less heat than shortening contractions (concentric), when the muscle performs work [2]. Similarly, walking uphill required almost double the energy compared to walking downhill [129]. These observations were later confirmed by the “negative Fenn effect”, which demonstrated that eccentric contractions require less energy than concentric and isometric contractions [1]. Despite these findings, Hill [16] noted that he primarily focused on positive mechanical work (concentric contractions), overlooking the importance of negative mechanical work, where energy is absorbed during muscular lengthening. Eccentric exercise is unique in that it demands significantly less energy than concentric exercise, yet resting energy expenditure remains elevated for several days after eccentric exercise.

**Figure 3:**
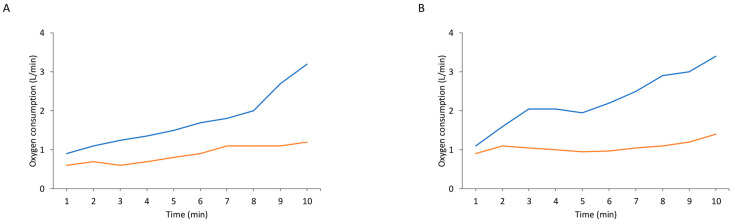
Both the landmark original investigation ((**A**); data from [130]) and the replication investigation ((**B**); data from [138]) showed that less oxygen was consumed during resisting bicycling (eccentric exercise; orange line) compared with forward pedalling (concentric exercise; blue line).

The distinct energy demands of eccentric exercise were first highlighted by Abbott et al. in a study that used an innovative experimental setup consisting of two back-to-back bicycles connected by a single chain. Their findings revealed that eccentric muscle contractions (backwards cycling) required significantly less energy than concentric contractions (forward cycling) [130] (Figure 3A). This surprising discovery led researchers to hypothesize that during muscle lengthening, the work absorbed by the contractile muscle reduces oxygen consumption [14,130]. Specifically, due to the fact that the heat or mechanical energy that is dissipated during eccentric (lengthening) muscle contraction does not reappear, it was suggested that certain chemical reactions might reverse during eccentric contractions, potentially resynthesizing ATP and lowering energy expenditure [131,132]. This concept, known as the exothermic energy contraction theory, which is no longer considered valid, provides a bioenergetic explanation for the lower energy cost of eccentric versus concentric contractions.

Abbott and colleagues attributed the reduced oxygen consumption during eccentric exercise to lower muscle fiber oxygen requirement and/or excitation frequency compared to concentric exercise [130]. They also suggested that during lengthening contractions under load, muscles behave like a wire stretched beyond their elastic limit, but without permanent deformation [133]. This reversible stretching stores energy that is released during the subsequent shortening phase of the contraction [37]. However, calculating energy turnover during muscle shortening proved difficult because researchers had to consider both the energy produced by the muscle and the energy released by its elastic components [132]. This concept, involving muscle tissue’s elastic network, was later revised by Huxley’s sliding-filament theory, which attributes force generation to cross-bridges between overlapping actin and myosin filaments [134,135,136,137].

Abbott’s experimental design [130] was replicated decades later using advanced technology, with similar results, that is, the energy cost of eccentric exercise remained significantly lower than concentric exercise [138] (Figure 3B). Proposed mechanisms for this include the winding filament theory of the protein titin in sarcomeres [139] and the theory of mechanical detachment of actin-myosin bound [140,141].

**Figure 4:**
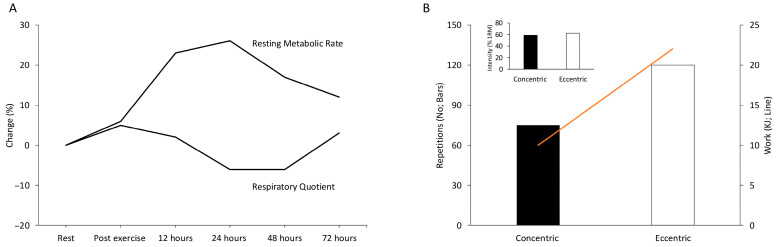
(**A**) Resting metabolic rate and respiratory quotient up to 72 h after a single bout of eccentric exercise (data from [148]). (**B**) The number of repetitions (bars) and total work (orange line) were substantially greater during eccentric than concentric isokinetic exercise performed to exhaustion at the same relative intensity (embedded figure) (data from [147]).

Muscle contraction velocity is critical to force production during eccentric contractions [3,142]. As velocity increases, the formation of cross-bridges decreases, and their detachment rate increases [143,144]. Interestingly, force increases with velocity in eccentric contractions, reaching a plateau higher than the maximal force produced during isometric muscle contraction [3,145,146]. Huxley’s model suggests that at high eccentric velocities, fewer myosin heads bind to actin, and the cross-bridges are stretched to a point of detachment, reattaching at longer sarcomere lengths without ATP consumption [135,137]. This mechanism explains the higher force production at lower energy cost during eccentric contractions [25,140,141,146]. In a recent study by our group, submaximal eccentric exercise demonstrated lower energy demands while producing greater work compared to concentric exercise [147]. Specifically, during submaximal isokinetic eccentric exercise to exhaustion, participants performed 30% more contractions and achieved 30% higher total work output compared to concentric exercise [147] (Figure 4B).

During eccentric contraction, the overlap between actin and myosin filaments decreases, yet force production increases up to a plateau, emphasizing the crucial role of structural scaffolds during eccentric contraction [44,149]. The reduced energy requirement during eccentric contraction is partly due to the increased passive tension provided by titin, a protein that functions as an internal spring, storing and releasing elastic energy [139,150,151]. According to the winding filament theory, titin’s N2A and PEVK segments bind to actin and wind around it during eccentric contractions, a process regulated by calcium release [139,152]. This theory helps explain both the reduced ATP requirements during eccentric exercise and the increased force production [153,154]. Moreover, it could be hypothesized that a possible reason for the lower energy expenditure during eccentric muscle contractions could be attributed to the ability of the neuromuscular system to operate with higher values of both muscle and tendon gearing, which has been suggested to play a role in determining the energy cost of movement [155]. The observation that the pennate muscles operate with a high gear ratio during eccentric contractions (leading to reduced rate of fascicle lengthening relative to the whole muscle), could be a way to mitigate the potential risk of muscle damage in pennate muscles [156].

The ability of eccentric exercise to generate higher force compared to concentric and isometric muscle contractions, is accompanied by lower energy demands. While current theories provide a strong foundation for understanding these unique bioenergetics properties of eccentric exercise, a deeper exploration of titin’s role in eccentric force production may offer new insights into muscle adaptation and training strategies.

### 4.2. Energy Expenditure During the Recovery Period (Days) Post Eccentric Exercise

While eccentric exercise requires less energy than concentric exercise, resting energy expenditure remains elevated in the days following eccentric exercise. The mechanisms behind this are distinct. Muscle repair following eccentric exercise-induced muscle damage is a slow, multi-phase process involving inflammation, necrosis of damaged fibers, and the proliferation and differentiation of satellite cells [157]. These repair processes are energy-intensive, contributing to the elevated resting energy expenditure observed for up to 72 h after eccentric exercise [148,158] (Figure 4A).

Protein synthesis, which accounts for about 20% of resting metabolic rate, plays a significant role in this increased energy expenditure [159]. The stimulus for hypertrophy following eccentric contractions [160] and the muscle repair processes also contribute to the higher energy demands during recovery [161]. Additionally, the lower respiratory quotient observed after eccentric exercise suggests greater fat oxidation [148] (Figure 4A). This could be due to increased fatty acid hydrolysis in damaged muscle membranes [162] and the dysfunction of glucose transport systems caused by muscle damage [163,164].

### 4.3. Take Home Message

Eccentric exercise, despite its lower energy demands during activity, leads to a prolonged elevation in resting energy expenditure during recovery, driven by energy-intensive muscle repair and adaptation processes. The prolonged increase in energy expenditure following eccentric exercise underscores its potential for metabolic conditioning and rehabilitation programs. Future experimental designs should focus on optimal intervention protocols to sustain these metabolic benefits even beyond the adaptations that occur over time in response to repeated eccentric exercise.

## 5. Repeated Sessions of Eccentric Exercise

### 5.1. The Repeated Bout Effect Phenomenon

**Figure 5:**
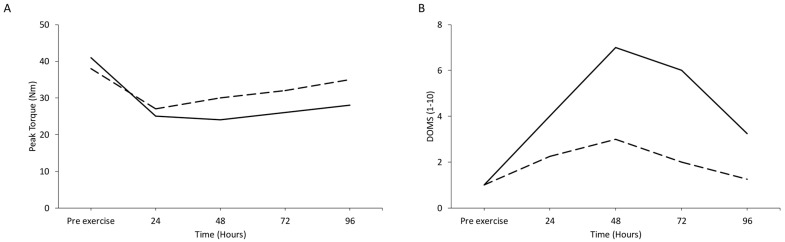
Repeated-bout effect after eccentric exercise, illustrated by changes in (**A**) peak torque and (**B**) delayed-onset muscle soreness (DOMS). Solid line: first eccentric exercise session; dashed line: second eccentric exercise session (data from [171]).

Compelling evidence supports the existence of cellular memory within muscle fibers [165], indicating that stem cells in muscles retain a memory of their in vivo environment in response to exercise. This phenomenon, known as the *repeated bout effect* was initially observed in two studies, where a second session of either isometric exercise [166] or downhill running [167] resulted in significantly reduced alterations in biomarkers of muscle damage (Figure 5A,B). One possible mechanism behind this effect is the structural adaptation of muscle tissue, including the strengthening of surrounding connective tissue in response to eccentric exercise [168,169,170].

The adaptation following muscle-damaging exercise involves a combination of neural, connective tissue, cellular, and inflammatory responses [172]. Neural adaptation includes a shift in motor unit recruitment from fast- to slow-twitch muscle fibers, which are more resistant to damage. Cellular adaptation likely involves an increase in the number of sarcomeres arranged in series, which reduces strain on sarcomeres during subsequent exercise bouts and helps prevent further damage [173].

Inflammatory processes are also thought to play a role, potentially triggered by oxidative stress, as similar changes in oxidative stress and muscle damage biomarkers have been observed after a second exercise session [174]. Additionally, the repeated bout effect may be due to changes in pain-related molecules, such as reduced expression of soreness-related mRNA [175]. Adaptations in muscle connective tissue, evidenced by decreased collagen genes expression following a second session of eccentric exercise [176], further contribute to this phenomenon.

Lower extracellular matrix mRNA levels have been found following repeated electrically stimulated isometric contractions, possibly due to a complex reorganization of gene expression that provides protection against future injury [177]. However, the precise role of matrix remodeling in injury prevention remains not well defined [86]. It is clear that no single mechanism can fully explain the repeated bout effect, as it likely results from the interplay of multiple mechanisms, some of which may still be undiscovered.

In summary of the above mentioned mechanisms, it could be suggested that the repeated bout effect could arise from: (i) neural adaptations which may include enhanced motor unit recruitment and synchronization, improved coordination, and greater contribution of synergist muscles, thereby distributing the load more evenly across fibers; (ii) mechanical and architectural changes, such as increases in fascicle length, sarcomeres in series, and connective tissue stiffness, which are proposed to shift the operating range of the muscle to more optimal lengths and enhance its ability to resist stretch-induced disruption; (iii) cellular adaptations, strengthening of the cytoskeleton and extracellular matrix and improvements in excitation–contraction coupling, which may confer greater structural stability during subsequent eccentric loads. In addition, repeated eccentric bouts appear to blunt the magnitude of the inflammatory and oxidative stress responses, attenuating leukocyte infiltration and altering cytokine and redox signalling, thereby limiting secondary damage and accelerating recovery.

The repeated bout effect is a powerful protective adaptation that highlights the muscle’s remarkable ability to “learn” from prior eccentric exercise and better resist damage in subsequent sessions. This has significant implications for training and rehabilitation, suggesting that controlled exposure to eccentric contractions can enhance muscle resilience while minimizing excessive muscle damage. Moreover, the repeated bout effect renders eccentric exercise an ideal experimental model, where a single acute session triggers prolonged changes of muscle damage, lasting up to three days [178], providing a sufficient time window to capture peak alterations, a condition followed by substantial adaptations after the second session [107].

Exercise-induced inflammation plays a critical role in driving adaptations, as cellular adaptations are impaired when anti-inflammatory agents are administered alongside exercise [111,112]. Paradoxically, the beneficial anti-inflammatory effect of exercise appears to be a result of the muscle microdamage caused by the exercise itself [179]. However, the initial inflammation is either greatly reduced or absent during repeated bouts of exercise, as demonstrated by the repeated bout effect [180,181].

Based on these findings, a novel exercise model has been proposed to overcome the plateau caused by the repeated bout effect [171]. This model alternates cycles of “micro-damaging” eccentric exercise with periods of “non-damaging” concentric exercise (e.g., 4 weeks of eccentric-only exercise followed by 4 weeks of concentric-only exercise) [171]. As expected, the initial eccentric exercise caused significant alterations in muscle micro-damage and inflammation biomarkers, which diminished after four weeks, indicating the onset of the repeated bout effect. The subsequent 4-week period of concentric-only exercise was sufficient to return the muscle to its unaccustomed state, effectively reintroducing acute muscle microdamage and inflammation while nearly eliminating the repeated bout effect [171]. This alternating contraction scheme allowed eccentric exercise to regain its “unaccustomed” nature, which is essential for maintaining cycles of muscle microdamage. These repetitive cycles are intended to surpass the adaptational plateau, with the microdamage hypothesized to trigger the muscle’s adaptive response [174,182].

This alternating exercise scheme could be particularly beneficial for individuals experiencing *inflammaging*, a condition characterized by chronic low-grade inflammation in older adults, marked by slightly elevated cytokine production [183,184,185]. Inflammaging impairs the body’s ability to manage degenerative processes related to inflammation, contributing to sarcopenia, a condition that reduces functionality and significantly diminishes quality of life in the elderly [186]. The proposed alternating cycles of muscle damage and inflammation may continuously activate the body’s anti-inflammatory mechanisms, offering a potentially effective strategy to combat inflammaging [171].

The alternating eccentric exercise model offers an innovative approach to overcoming the adaptational plateau induced by the repeated bout effect. By systematically alternating between eccentric and concentric exercise phases, this model can sustain beneficial cycles of muscle microdamage, inflammation, and subsequent adaptation. This approach is particularly promising for aging populations, as it may counteract inflammaging and support muscle health. Further research should explore the long-term implications of this model for improving muscle function and overall metabolic health, especially in clinical populations.

### 5.2. Chronic Eccentric Exercise: Eccentric Exercise per se Does Not Induce Muscle Damage

Chronic eccentric exercise was first studied in an experimental setting, where it was shown to increase muscle strength, reduce soreness, and enhance asynchronous motor units recruitment [18]. This desynchronization of motor units was linked to muscle hypertrophy [187]. A critical finding from this study was that eccentric exercise is not inherently associated with muscle damage. However, due to its initial damaging nature, it was recommended to avoid eccentric exercise at the beginning of a training intervention [18].

Later research revealed that chronic eccentric exercise could gradually reduce muscle damage, as demonstrated by decreased severity of myofibrillar injuries [188]. It is well-established using electrical stimulation that strength output is much greater during eccentric compared to other types of muscle contraction [3,18,189,190], leading to the consensus that eccentric exercise induces greater muscle size and strength increases [191]. However, contrary to this belief, some studies have found that eccentric and concentric muscle contractions during conventional resistance training can produce comparable muscle hypertrophy [49,192] and strength gains during isokinetic exercise [53,171,193] or during eccentric cycling ergometer [194,195] when exercise intensity and volume are matched.

During strength training that focuses on either pure eccentric or concentric muscle contractions (e.g., using isokinetic dynamometry), the principle of training specificity becomes evident. Specifically, eccentric strength may increase more following eccentric training, and vice versa for concentric training [53,182,188,196]. However, when strength is assessed isometrically, gains are similar between both training modes [53,182].

Early studies recommended the gradual introduction of eccentric contractions into training regimens to avoid the detrimental effects of initial eccentric exercise on performance and soreness [18]. Interestingly, research later revealed that muscle damage is not a prerequisite for exercise adaptations [197,198]. Chronic eccentric exercise can induce similar improvements in performance, even when muscle damage is minimized or absent [194,195,199], suggesting that muscle damage is neither a limitation nor a requirement for training adaptations [191,200].

It is evident that eccentric training ultimately leads to adaptations similar to those of other forms of exercise. Since neither eccentric nor concentric contractions consistently show clear superiority in muscle mass or strength gains, researchers suggest that combining both may be more effective for muscle growth [192,201,202]. This idea is supported by a meta-analysis that found that combining concentric and eccentric muscle actions produces the greatest strength increases compared to either concentric-only or eccentric-only training [203].

Eccentric muscle contraction-based training provides an optimal form of physical activity by combining lower energy expenditure and perceived exertion with positive health-related outcomes [182,194]. This type of training is especially beneficial for individuals with limited physical capacity, as eccentric exercise demands less energy than concentric exercise when performed at the same perceived exertion during bicycling [130] and stair ascending/descending [204]. For example, in elderly individuals with chronic cardiovascular disease, stair descending has been shown to be a pleasant and mild form of exercise that is easy to follow, while offering similar gains in muscle strength compared to the more strenuous stair ascending exercise [204].

**Figure 6:**
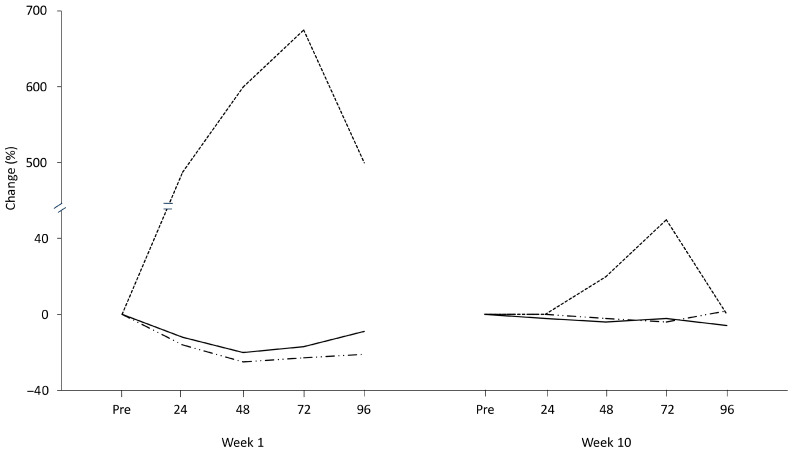
Changes in muscle-damage indicators across a 10-week eccentric training intervention. In the first week, eccentric exercise induced significant changes in range of motion (ROM; solid line), isometric peak torque (dashed line with markers), and delayed-onset muscle soreness (DOMS; dashed line), indicating substantial muscle damage. By the last training phase (week 10), these responses were no longer evident, showing that chronic eccentric exercise per se does not worsen these muscle-damage indicators. These findings run contrary to the common view that eccentric exercise is inherently damaging (data from [53]).

There is an ongoing debate regarding eccentric exercise. While it is known to cause muscle damage, it is also recognized as a mode of physical activity that can enhance health and performance with lower energy requirements compared to other forms of exercise. The link between eccentric exercise and muscle damage has been highlighted in studies using isokinetic dynamometers, which allow for pure eccentric contractions at maximal intensity, making muscles particularly vulnerable to injury. However, the progressive reduction in muscle damage following repeated sessions of eccentric exercise suggests that it is the unaccustomed nature of eccentric contractions, rather than the eccentric contractions themselves, that primarily contributes to muscle damage [53] (Figure 6).

### 5.3. Take Home Message

The ancient Roman god *Janus* symbolized the progress from one condition to another, since he could see into the past with one face and into the future with the other. In a Janus-like manner, it could be suggested that eccentric exercise leads from one condition, that is, muscle damage in the acute phase, to another condition, that is, diminishes muscle damage and promotes muscle adaptation in response to continued exposure to eccentric exercise. However, despite the fact that eccentric training holds unique muscle adaptation characteristics, neither eccentric nor concentric training alone seems to be superior. It could be suggested that optimal programming strategies that balance eccentric and concentric training may offer the most effective approach to optimizing muscle growth and performance.

From a research perspective, in future studies the dose–response relationships (intensity, volume, frequency) should be clarified for maximizing the beneficial adaptations while minimizing the excessive damage. Moreover, the relative contribution of neural, architectural, connective tissue, and molecular factors to the repeated bout effect should be elucidated. Finally, more work is needed to integrate eccentric exercise with nutritional and recovery strategies, so that its unique properties can be exploited safely and effectively in both health and performance frameworks.

## 6. Architectural and Morphological Characteristics of Adaptation to Eccentric Training

While eccentric training induces many of the same physiological adaptations as concentric training, the two differ markedly in energy demands and motor recruitment. Eccentric muscle contractions have a lower metabolic energy cost [138], recruit fewer motor units [205], and generate greater tension per active motor unit [3,17,18] than concentric contractions. Over time, eccentric versus concentric training also yields distinct muscle architectural and morphological changes in muscles.

**Figure 7:**
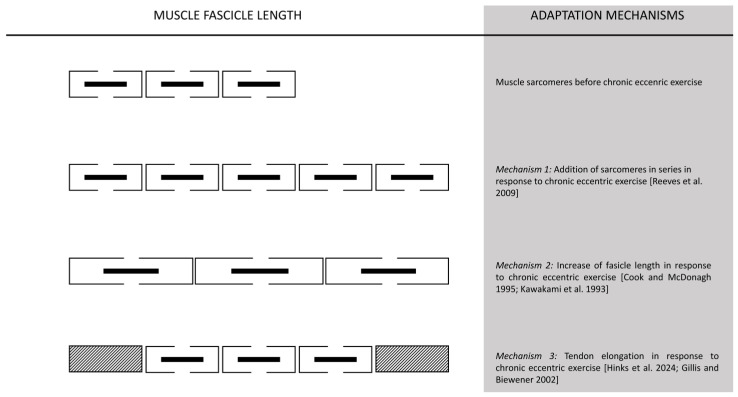
Proposed simplified mechanisms for muscle fascicle length adaptations in response to chronic eccentric exercise. Cook and McDonagh 1995 [189]; Gillis and Biewener 2002 [220]; Hinks et al. 2024 [221]; Kawakami et al. 1993 [216]; Reeves et al. 2009 [217].

Increases in fascicle length assessed at rest after chronic eccentric training are attributed to two key factors: the elongation of each sarcomere (short-term alteration) [206,207] and the addition of sarcomeres in series (long-term adaptation) [208,209] (Figure 7). Early work—using the most advanced methods available at the time—suggested that sarcomeres are added near muscle fiber ends [209], potentially originating at the Z-disc [210]. In humans, chronic high-intensity training has been associated with structural reorganization, including evidence consistent with increased sarcomere proliferation at the Z-band and a broader distribution of sarcomere lengths in type II muscle fibers—adaptations commonly interpreted as responses to the high mechanical demands and myofibrillar disruption induced by eccentric contractions [188,211]. It has been proposed that eccentric training also increases fascicle length, likely due to the addition of sarcomeres in series [212,213,214], while concentric training increases pennation angle, which is thought to result from the addition of sarcomeres in parallel [215,216,217]. Notably, different muscles may respond differently to the same type of muscle contraction [218,219].

Contemporary procedures in humans that directly assess sarcomere behavior—such as endoscopic sarcomere length measurements—also report increased fascicle length in response to eccentric training performed over a larger range of motion [222,223]. Notably, these studies appear to diverge on mechanism, since one attributed the fascicle length increase primarily to longer sarcomeres (i.e., increased sarcomere length; [223]), whereas the other favored an in-series increase in sarcomere number [222]. This discrepancy underscores the need to directly measure the in-series sarcomere number to clarify the drivers of longitudinal fiber growth [224]. Taken together, these findings are consistent with the broader view that muscles adapt to the high tension of eccentric contractions (a mechanical signal) by regulating protein synthesis (a biochemical response) [206,207,208,209].

Animal studies further support the addition of sarcomeres in series after eccentric contractions [209,214,218,225,226,227]. Recent syntheses, however, highlight unresolved issues regarding series sarcomere number changes. Based on a subset of animal studies, it has been proposed that electrically stimulated eccentric training does not increase series sarcomere number [224]. In contrast, considering animal data across a broader set of exercise modalities (i.e., downhill running and dynamometry with electrical muscle stimulation for all muscles investigated) may support the increase in series sarcomere number [228]. Moreover, current evidence suggests eccentric training is not uniquely required for sarcomere addition in skeletal muscle [224]. For example, isometric training at long muscle lengths can substantially increase fascicle length [229]. Interestingly, increased sarcomere number in series can rapidly revert toward baseline with the return to normal activity after long-duration immobilization [208].

Both eccentric and concentric training produce architectural changes in muscles across age groups [215,217,230]. Although similar whole muscle hypertrophy has been observed after both modes [192], they promote distinct regional hypertrophic patterns [215]. Eccentric training tends to favor hypertrophy in the distal regions of muscle, while concentric training promotes hypertrophy in the mid-belly region [215,231,232,233,234].

In summary, eccentric training drives architectural and morphological adaptations in muscle that differ from those of concentric training. Both eccentric and concentric training promote hypertrophy, but with distinct regional specificity, implying that balanced programs incorporating both contraction types may optimize muscle architecture and function. Given its lower metabolic cost, eccentric training offers an efficient strategy to improve performance across diverse populations. These findings highlight the importance of tailoring training programs to leverage the unique benefits of eccentric training, including athletes seeking high-force adaptations and individuals with limited energy capacity who require effective, lower-energy-demand exercise options.

### 6.1. Challenges and Insights from Animal Models

The findings of Lynn and Morgan [226], regarding increased sarcomeres after eccentric training in rats, have been questioned due to methodological flaws such as the absence of force measurement, lack of force control, and missing joint kinematics. These omissions prevent a direct link between the increase in sarcomeres in series and eccentric contractions [219]. In contrast, a 12-week study involving controlled eccentric muscle training using electrical stimulation in rabbits’ dorsiflexor muscles showed little or no increase in sarcomere number [219]. A more recent study found that 2 weeks of eccentric training increased the number of sarcomeres in series and torque production in young rats, while older rats exhibited blunted sarcomerogenesis and reduced torque [221].

Animal data should be interpreted cautiously due to possible methodological limitations that could lead to misleading conclusions. For example, rats show different training adaptations across muscle groups [218], and their knee extensor muscles perform eccentric contractions whether running uphill or downhill [220]. Additionally, differences in muscle architectural remodeling mechanisms between rats and humans must be considered. Rats show greater increases in ERK and p38 MAPKs phosphorylation [235,236], whereas humans exhibit greater MAPK activation following eccentric versus concentric training [215].

It is clear that despite animal models providing valuable insights into muscle adaptation mechanisms, their findings should be interpreted with caution due to methodological limitations and species-specific differences. While some studies support the idea that eccentric training leads to an increased number of sarcomeres in series, others show conflicting results, highlighting the complexity of muscle adaptation. The discrepancies between animal and human data create uncertainty regarding skeletal muscle adaptation mechanisms, which indicates that may not be appropriate to directly extrapolate animal data to human training and rehabilitation protocols.

### 6.2. Long-Term Muscle Adaptations

A recent study showed that chronic concentric training decreased the number of sarcomeres in series, whereas concurrent eccentric training did not induce sarcomerogenesis [237]. However, sarcomere addition in response to eccentric training appears to be short-lived; sarcomere numbers increase in the first week of training but return to baseline by the eighth [238]. This suggests that tendon elongation, rather than sarcomere addition, might contribute to muscle fiber lengthening [237,238] (Figure 7). Moreover, it has been proposed that eccentric training may lead to an increase in sarcomere length rather than an increase in the number of sarcomeres in series [223]. Specifically, individual sarcomeres length was found to increase by 17%, suggesting that sarcomerogenesis may not be necessary to handle the high force output during eccentric muscle contraction [223].

It is important to note that the measurement of sarcomeres in series using a single biopsy provides only a spaciotemporal “snapshot” of the entire muscle, potentially missing essential information about sarcomere adaptation. Both eccentric and concentric training, however, have been found to produce similar anabolic responses, including increases in mRNA and decreases in myostatin mRNA [239]. Additionally, mTORC1 signaling appears to be unaffected by the type of contraction [240]. This suggests that hypertrophy may be driven more by an increase in the number of actin and myosin molecules within each sarcomere than by changes in the number of sarcomeres as functional units. The unique aspects of neuromuscular control in eccentric versus concentric muscle contractions were first described by Komi & Viitasalo [241], who observed an increase in EMG recording during low-force isometric contraction only following muscle-damaging eccentric exercise. Since then, numerous studies have documented distinct neuromuscular control strategies between eccentric and concentric muscle contractions.

### 6.3. Take Home Message

The long-term adaptations to eccentric training are more complex than previously thought, with sarcomere addition appearing to be a transient phenomenon rather than a sustained structural change. Instead of increasing the number of sarcomeres in series, eccentric training may lead to longer sarcomeres or tendon elongation, which could explain the muscle’s ability to withstand high mechanical loads. Researchers should focus on exploring the distinct muscle adaptation mechanisms underlying different types of muscle contractions, as both eccentric and concentric training elicit similar hypertrophic and molecular responses.

## 7. Neural Responses and Adaptations to Eccentric Exercise

**Figure 8:**
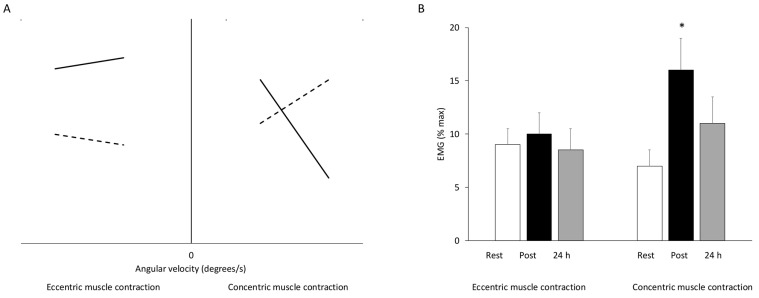
(**A**) Although maximum torque is higher during eccentric than concentric contractions (continuous line), amplitude of EMG, that reflects muscle activation, is lower during eccentric than concentric contraction (dashed line) (adopted from [247], used with permission. © 1985, American Physiological Society). (**B**) Triceps brachii EMG during elbow flexion concentric and eccentric exercise at rest and after exercise (adopted from [248], used with permission. © 2007, American Physiological Society). * significant different from rest condition (*p* < 0.05).

The shift in focus from mechanical output to neural drive in voluntary eccentric contractions began when early experiments showed that, for equal or greater force, the surface EMG signal was smaller during lengthening than during shortening actions [242]. These early reports were confirmed in later years, when evidence showed that people can produce large torques with lower surface EMG during eccentric contractions (Figure 8A). This indicates that eccentric contractions are controlled differently by the nervous system and helped establish the neural perspective that followed [243,244,245,246]. Later, Enoka argued that lengthening contractions require distinct activation strategies, shifting the focus from the amount of drive the muscle receives to the manner in which it is driven [247].

Currently, enhanced cortical excitability and spinal inhibition are believed to be the primary mechanisms underpinning the reduced motor activity during eccentric compared to concentric muscle contractions [249,250,251]. It has been suggested that during eccentric contractions, a combination of central and peripheral factors biases control toward a smooth and stable lengthening phase, which matches the sharp early drop in performance often observed [250]. Consistent with this notion, it can be hypothesized that this condition contributed to the reduced rate of force development observed two days after an ecologically performed eccentric exercise [252]. Moreover, the earlier onset of cortical activation during eccentric versus concentric contractions [253], has been attributed to greater processing of feedback signals needed to plan a more complex movement, and/or to implement a different control strategy (e.g., motor unit recruitment) [254,255].

### 7.1. The Effect of Acute Eccentric Exercise on Neural Control

The eccentric muscle contractions, which involve a mix of central and peripheral factors [255], together with the close proximity of nerves and muscle fibers at the neuromuscular junction (NMJ), render nerves susceptible to damage in response to unaccustomed exercise [256,257,258]. Indeed, work focusing on the NMJ has shown that eccentric exercise in dystrophic animal models causes disruptions in nerve-muscle connection and slower recovery compared with healthy animals [259]. In healthy adults, standardized lengthening contractions cause substantial but reversible neuromuscular disruption [260,261], indicating that eccentric exercise can reshape pre- and post-synaptic features of the NMJ [262].

In response to exercise-induced muscle damage, the reduced neuromuscular performance may be at least partly underpinned by impaired sarcolemmal action potential conduction velocity [263] and transient changes (e.g., for 24 h) in central nervous system activity [264]. After eccentric exercise, significant decreases in sciatic nerve motor nerve conduction velocity, as well as narrowing of its axons and myelin sheath, have been reported [256,265]. Additionally, after a bout of unaccustomed maximal lengthening contractions, studies combining motor nerve stimulation and TMS [266] suggest that impaired signals from the brain and spinal cord may contribute to reductions in voluntary activation, indicating a neural contribution to the reduction in maximal voluntary torque-producing capacity [267,268,269].

### 7.2. Transcranial Magnetic Stimulation (TMS)

The use of transcranial magnetic stimulation (TMS) has enabled researchers to obtain more rigorous and precise data on motor-evoked potentials and corticospinal excitability. TMS can painlessly and selectively probe motor cortex excitability via motor-evoked potentials, allowing a more accurate assessment of how effectively neural commands travel from brain to muscle [269,270,271]. However, results obtained with TMS require interpretation and complementary measures, because TMS responses vary with the task being performed, while dynamic eccentric actions may amplify this variability [270,271,272,273].

### 7.3. Neural Adaptations to Chronic Eccentric Exercise

In response to chronic conventional resistance exercise, neural adaptations may occur at the level of the motor cortex, spinal cord, and/or neuromuscular junction [253,274], which is evident from the disproportionate increase in muscle force compared with muscle size during the initial stages of training [254]. Skeletal muscle may also adapt after being exposed to a second eccentric exercise stressor that initially caused muscle damage, a phenomenon known as the repeated bout effect (RBE) [178,182,275].

Among the multiple factors involved in RBE are alterations in voluntary activation and attenuation of motor unit behavior [266,276,277], suggesting modulation of spinal and supraspinal inputs to the motoneuron pool. These adaptations may include increased motor unit activity relative to the force produced, increased synchrony of motor unit firing, and/or altered recruitment patterns [278]. Several investigators have reported a shift in the frequency content of the EMG during performance of the repeated bout, suggesting a concurrent increase and decrease in slow- and fast-motor unit activity, respectively [268,279].

Increased reliance on slow motor units (i.e., reduced EMG high-frequency content) may confer protection during the repeated bout by decreasing stress on susceptible fast-twitch fibers, and promoting a more even distribution of contractile stresses [60]. It has also been suggested that adaptations in the sarcolemma, such as slower action potential conduction velocity, may explain altered spectral frequency characteristics of the EMG rather than specific damage its fast-twitch fibers [280] (Figure 8B).

### 7.4. Cross-Educational Effect

Cross-transfer or cross-education is a phenomenon driven largely by neural adaptations, whereby strength increases in the contralateral limb following training of the ipsilateral (trained) limb [281,282,283]. Connolly et al. [284] were the first to examine the protective effects of a repeated bout effect that could transfer to the contralateral limb and found no evidence of such a cross-transfer. However, subsequent work (e.g., [268,285,286,287,288,289]) has generally supported the notion that the protective effects of a repeated bout can be transferred contralaterally.

It has been suggested that the contralateral repeated bout effect may be due to neural adaptations similar to those underpinning the cross-education effect, in which resistance training of one limb increases strength of the contralateral limb [268,290,291]. Additionally, it has been proposed that the contralateral repeated bout effect could also involve an inflammatory component, whereby an initial increase in the inflammatory-related transcription factor nuclear factor kappa–light-chain-enhancer of activated B cells (NF-JB) acts as an effector of an upstream mechanistic pathway that can be transferred to non-exercised contralateral muscles [289].

### 7.5. Eccentric Exercise and Proprioception

It has been clearly shown that eccentric muscle damage impairs proprioception, that is, it alters how people perceive joint position and force [292]. Unaccustomed eccentric loading has been found to transiently blunt proprioception for approximately 24–72 h, as evidenced by increased joint position matching errors, elevated thresholds for detecting passive movement, and a biased sense of force changes that diminish with familiarization [39,293,294,295]. During lengthening, the nervous system appears to downweight spindle afferent input to stabilise the negative work phase, producing lower EMG for the same torque and a depressed short-latency stretch reflex, consistent with tighter control [39,250,293,294,295,296]. By comparison, isometric and concentric tasks show smaller and shorter-lived proprioceptive effects, often reflecting a higher sense of effort, and these tend to resolve with training [275,297,298,299].

Later work shifted the focus to the time frame immediately preceding motion. Grabiner and Owings [300] showed that, even with identical starting conditions, muscle activity is lower just before an eccentric movement than before a concentric one, a planned “use-less-signal” preset that emerges before mechanics can play a role. In tasks with an enforced waiting period, eccentric trials show a small drop in drive from brain to muscle just before the Go signal, followed by a rapid rebound—a brief brake-then-release pattern. At the same time, the muscle sensors (spindles) and the short stretch reflex are dialled down during the waiting period preceding an upcoming stretch [301,302]. Collectively, anticipating lengthening under load appears to lead to a coordinated down-weighting of spindle inflow and outgoing drive so the onset of lengthening is smooth rather than irregular [301,302].

### 7.6. Take Home Message

Eccentric exercise is governed by a distinct neural control strategy compared to concentric exercise, characterized by lower EMG for the same or greater torque, enhanced cortical excitability, and increased spinal inhibition, alongside altered spindle input that can transiently impair proprioception and neuromuscular performance in response to unaccustomed stimulus. With repeated exposure, however, these acute disturbances give way to adaptive changes in motor unit recruitment, voluntary activation, and even cross-limb transfer, contributing to the repeated bout effect and improving motor control, protection from future stressors, and strength enhancements.

## 8. Exercise-Induced Muscle Damage, Mitochondria and Microstructural Adaptations

### 8.1. Acute Effects of Eccentric Exercise on Mitochondrial Function

**Figure 9:**
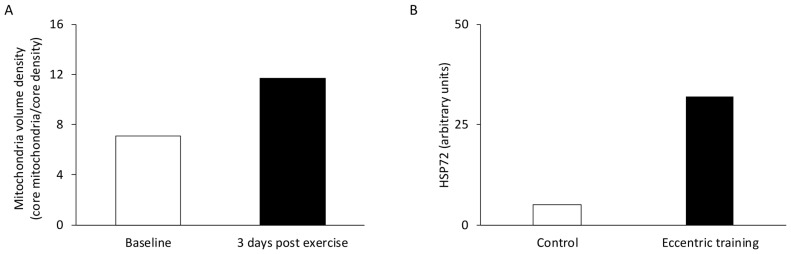
(**A**) Increased mitochondrial volume density in skeletal muscle after an acute bout of eccentric exercise, consistent with mitochondrial swelling (data from [188]). (**B**) Chronic eccentric exercise increases skeletal muscle heat shock protein 72 (HSP72) which may prevent mitochondrial injury (i.e., reduce mitochondrial calcium accumulation and reduce opening of the mitochondrial permeability transition pore) (data from [309]).

Resistance exercise imposes repetitive mechanical loading that stimulates mitochondrial remodeling through stress-activated signaling pathways [303,304]. Among resistance modalities, eccentric exercise causes pronounced ultrastructural disruption, including sarcomere disorganization and mitochondrial swelling [305]. Early investigations using muscle biopsies obtained 3 days after eccentric exercise reported that some muscle fibers contained swollen mitochondria, leading to increased mitochondrial volume density (Figure 9A; [305]). The swollen mitochondria were proposed to be a secondary effect of intracellular edema caused by the release of osmotically active ions [306]. More recent work using transmission electron microscope and stereological techniques confirmed that, following eccentric exercise, mitochondria can be grossly swollen and exhibit multiple ultrastructure defects, including few or absent cristae and reduced matrix electron density [307]. These alterations may affect both the opening of the mitochondrial permeability transition pore and direct mitochondrial membrane damage [308].

### 8.2. Oxidative Stress and Mitochondrial Function

The opening of the mitochondrial permeability transition pore has been attributed to excessive mitochondrial reactive oxygen species (ROS) production leading to redox disturbances [310]. Although ROS were long considered only harmful by-products of respiration that drive mitochondrial disorders and cell death, more recent evidence shows that mitochondria-derived ROS also participate in diverse cell signaling processes [311,312,313]. There is growing evidence that exercise-induced muscle damage is linked to ROS production [174,314]. Reactive oxygen species appear to be a critical component of the cellular stress–response cascade initiated by eccentric exercise, connecting mechanical strain, calcium dynamics, and redox-dependent signaling [315,316]. However, the role of mitochondrial-derived ROS in mitochondrial function remains debated. The high calcium concentrations reported after eccentric exercise [317,318], has been associated by some studies with impaired mitochondrial function [309,319] but not by others [320,321].

Eccentric exercise rapidly triggers mitochondrial quality-control pathways that help restore cellular homeostasis following mechanical stress [304,322,323]. Within 12–24 h of eccentric loading, mitochondrial fission increases, promoting segregation of damaged organelles [322,324,325]. Compensatory fusion rises to maintain network integrity and facilitate content mixing among intact mitochondria organelles [304,325,326]. Damaged mitochondria are subsequently removed via mitophagy, initiated within 24 h, indicating activation of autophagic flux [322,325,327]. Mitophagy intersects with autophagy, which clears protein aggregates and cytosolic debris generated by contraction-induced damage [328,329]. When ROS or calcium overload compromise lysosomal membranes, a secondary process termed lysophagy is engaged to recycle defective lysosomes and preserve degradative capacity [328,330].

### 8.3. Chronic Effects of Eccentric Training on Mitochondrial Function

The coordinated activation of these quality-control cascades typically restores mitochondrial respiration to pre-exercise levels within 72 h and primes subsequent biogenesis via AMPK–PGC-1α signaling [304,322,325,331]. With chronic exercise, mitophagy flux is reduced, reflecting a diminished need for organelle breakdown, while mitochondrial content and function increase [332]. Indeed, after a 12-wk training program, genes encoding mitochondrial repair and remodeling factors are upregulated [333]. Chronic eccentric exercise also upregulates heat shock protein 72, which may improve sarcoplasmic reticulum function (Figure 9B; [334]) and, in turn, reduce mitochondrial calcium accumulation and limit opening of permeability transition pore [335].

### 8.4. Microstructural and Sarcoplasmic Reticulum Adaptations in Response to Eccentric Exercise

Eccentric exercise induces pronounced alterations in the intracellular architecture of skeletal muscle, particularly sarcoplasmic reticulum (SR), transverse (T-) tubules, and cytoskeletal scaffolds that coordinate excitation–contraction coupling and mitochondrial metabolism [305,336,337]. The SR serves as the primary calcium reservoir, releasing Ca^2+^ via RyR1 and reclaiming it via SERCA1/2 during relaxation [316,338]. Following eccentric loading, microscopy reveals SR dilation, fragmentation, and partial uncoupling from T-tubules within 12–24 h, accompanied by swelling and structural disorganization at triadic junctions [6,22,23]. Quantitative morphometry indicates an up to 25% increase in SR volume density, reflecting transient calcium overload and osmotic stress rather than functional gain [6,22,23,108,337]. Oxidation of RyR1 can produce leaky channels and elevate cytosolic calcium by up to 40% above baseline [338,339]. This calcium leak promotes mitochondrial calcium uptake via the mitochondrial calcium uniporter, which can transiently stimulate dehydrogenases but also increase ROS emission [340,341]. The resulting feedback loop amplifies redox imbalance and mitochondrial stress [304,342]. Cytoskeletal proteins such as desmin, titin, and dystrophin, essential for sarcomeric alignment, are vulnerable to eccentric strain, while their partial disruption alters mitochondrial positioning and Ca^2+^ buffering [6,23,113]. With repeated bouts, however, the muscle remodels, that is, SERCA1/2 and calsequestrin expression increase, triadic junctions reinforce, and antioxidant defenses concentrate near SR–mitochondrial contact sites [304,322,336,342]. These adaptations enhance excitation–contraction coupling efficiency and protect mitochondria.

### 8.5. Take Home Message

Acute eccentric muscle-damaging exercise may couse disturbances in mitochondrial and sarcoplasmic reticulum structure, elevating calcium and reactive oxygen species and activating powerful quality-control pathways (e.g., fission–fusion, mitophagy, autophagy) to remove or repair damaged components. With repeated bouts, these same stress signals drive targeted remodeling of mitochondria, sarcoplasmic reticulum and cytoskeleton—upregulating repair genes, HSP72, calcium handling proteins and local antioxidant defenses—so that excitation–contraction coupling becomes more efficient and the muscle is better protected against future mechanical loads.

## 9. The Role of Connective Tissue in Exercise-Induced Muscle Damage

Seminal observations suggested that the basement membrane, a thin sheet of extracellular matrix, plays an important role in the orderly regeneration of damaged skeletal muscle [343]. Almost a century later, using animal muscle transplantation models, researchers proposed that injured skeletal muscle can fully regenerate only if the muscle’s basement membrane and extracellular matrix myofibers remain intact. These structures form an unchanged tube that provides the framework within which new myotubes and myofibers develop [88,91,344]. Conversely, the extracellular matrix is not a static structure, rather, in response to muscle injury, its major components are sequentially degraded and subsequently resynthesized as part of the myofiber repair process [345].

In contrast to the extensive research on skeletal muscle responses to eccentric exercise, relatively little attention has been paid to the dynamics of intramuscular connective tissue. However, early investigations suggested that DOMS following eccentric exercise may be related to disruption of connective tissue elements within the muscle and/or changes within the peri-muscular connective tissue [4,20,346]. Supporting these early findings, later studies demonstrated that stretch-induced injury causes disturbances not only in myofibers but also in the extracellular matrix in rat models [347], as well as after isokinetic eccentric exercise in humans [348]. Since tendons are viscoelastic tissues, where the degree of deformation depends on the level of loading and stiffness [349], it has been suggested that eccentric exercise may predispose tendons to damage due to their non-linear force–elongation relationship, potentially compromising tendon integrity [350].

An additional, relatively underexplored determinant of skeletal muscle resilience during eccentric loading may be the costamere–M-band axis. Costameres form specialized protein assemblies that mechanically link peripheral myofibrils to the sarcolemma and extracellular matrix and play a central role in lateral force transmission, thereby helping redistribute force when local sarcomeric disruption occurs [351,352]. This function may be particularly relevant in regions showing Z-line streaming or non-uniform myofibrillar strain after eccentric exercise [353]. In parallel, the M-band stabilizes the thick filament lattice and contributes to the structural integrity of the sarcomere, while its molecular organization appears to vary across fiber types [354,355]. Classic ultrastructural observations have shown fiber type–dependent differences in M-band/M-bridge appearance, with less distinct M-bridges in slower fibers and more prominent structures in faster fibers [356]. Taken together, these observations suggest that the costamere–M-band system may represent an underexplored site of adaptation to eccentric loading, potentially contributing to structural reinforcement and force redistribution. However, direct evidence that eccentric training specifically increases M-bridge number in human skeletal muscle remains limited [353,356].

Components of the connective tissue may also play a role in exercise-induced muscle damage. In a recent investigation, it was observed that in response to knee extensors, maximal eccentric exercise led to an increased stiffness of the deep fascia, which correlate with the magnitude of DOMS, indicating the hypothesized crucial role of fascia in the origin of DOMS [357]. Aponeuroses, on the other hand, are connective tissue sheets located on the surface of pennate muscles. They form a continuous structure with the external tendons and, like the tendons, transmit forces and length changes from the muscle fascicles to the skeleton [358]. It was observed that, in response to intense eccentric loading (i.e., high force, low velocity), incisions in aponeuroses reduce muscle gearing, that is, the ratio of whole muscle velocity to fascicle velocity, by altering the relationship between gearing and force [359].

**Figure 10:**
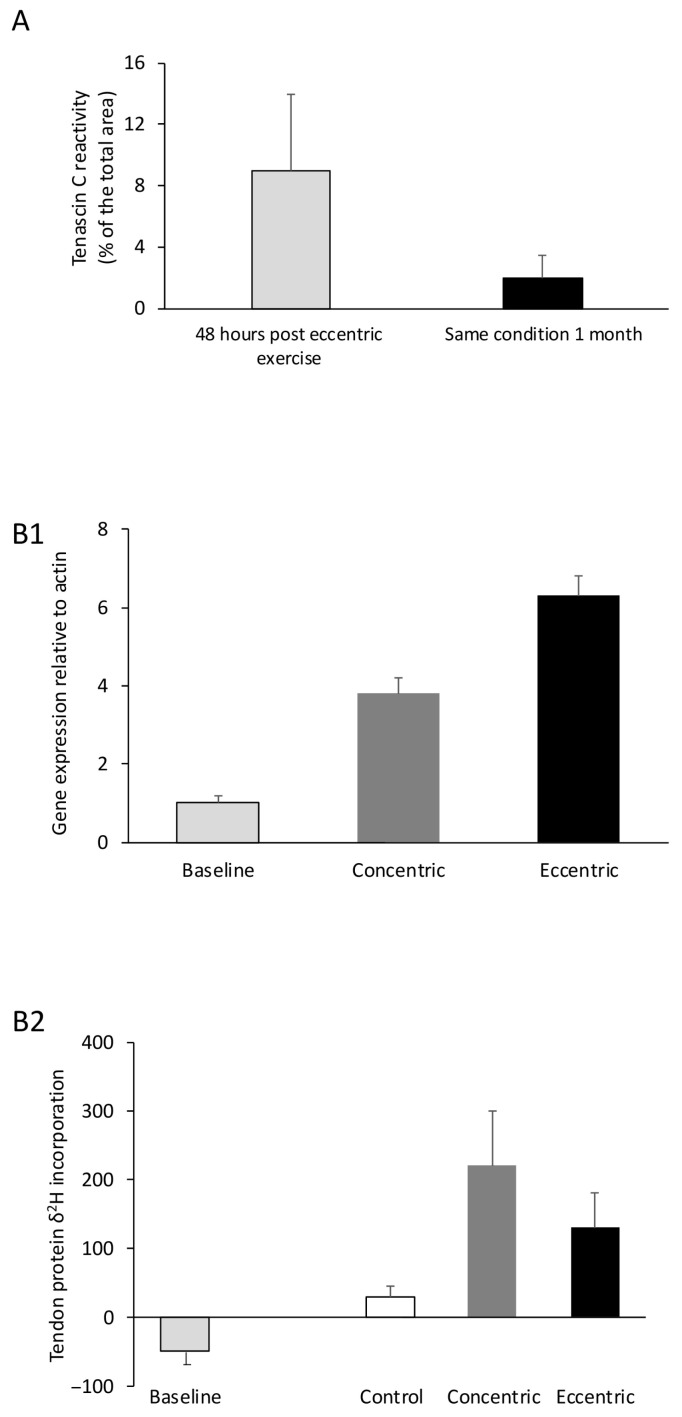
Tendon response to eccentric exercise. (**A**) Tenascin C (extracellular-matrix glycoprotein) in skeletal muscle 48 h after a single eccentric exercise bout (gray bar) and at the same time point 1 month later (black bar) (data from [177]). (**B1**) Expression of extracellular-matrix-related growth factor genes in tendon after 4-week eccentric and concentric training (data from [362]). (**B2**) Tendon protein δ2H incorporation after 4 weeks of eccentric or concentric training in tendon tissue (data from [362]).

The adaptation of muscle connective tissue to exercise is complex, since a matrix anabolic phase occurs later in the regeneration process, it is preceded by an earlier phase characterized by matrix de-adhesion and disassembly [177]. Regarding eccentric loading, despite its initially disruptive nature on connective tissue, repeated exercise sessions initiate adaptive processes in tendons and the extracellular matrix, leading to increased tendons stiffness [360]. This adaptation may enhance the rate of force development and reduced shortening of the in-series contractile component [361]. Indeed, alterations in the muscle’s extracellular matrix after an initial eccentric exercise bout are thought to contribute to early de-adhesion and successive remodeling of the extracellular matrix, thereby protecting the muscle against injury during subsequent exercise sessions [177] (Figure 10A).

Chronic training with heavy eccentric loading has been shown to increase tendon stiffness, though without evidence of tendon hypertrophy [363]. Based on animal experiments, it has been speculated that heavier loads during high-intensity eccentric training might serve as a more potent stimulus for tendon hypertrophy [364,365]. Interestingly, chronic eccentric exercise in humans appears to induce greater tendon remodeling at the gene expression level compared to chronic concentric exercise, which did not translate to differences in tendon protein turnover, resulting in similar increases in protein synthesis between the two training modalities [362] (Figure 10Β1,B2). Likewise, studies comparing pure concentric and pure eccentric resistance exercise reported similar adaptations in tendon mechanical properties [366], and tendon hypertrophy [367]. These findings reinforce the notion that the cellular and tissue response of healthy tendon is “blind” to the type of muscle action producing the strain. It has been suggested that compared to contractile elements, connective tissue requires prolonged high-intensity exercise loading to elicit robust changes in size and mechanical properties [368,369,370,371].

The mechanical adaptation of connective tissue is thought to arise from enhanced collagen turnover and structural reorganization of fibrillar networks [370,372]. Importantly, such changes were functionally beneficial, improving force transmission and joint stability. At the molecular level, increased expression of collagen genes such as 1A1 and 3A1 and TGF-b1 mRNA has been observed up to 8 days after eccentric exercise [373]. According to the authors, this upregulation indicates ongoing extracellular matrix remodeling during the recovery period [373]. Moreover, indirect observations highlight the role of inflammation as an important mediator for collagen turnover and tendon adaptation in response to eccentric exercise. Specifically, blocking inflammation in humans during acute exercise completely abolished the rise in collagen synthesis commonly seen in response to exercise [374]. Conversely, infusion of IL-6 into peritendinous tissue at rest stimulated an increase in collagen synthesis [375].

It has been highlighted that methodological challenges make research on tendon damage, recovery and adaptation in response to exercise a tricky task and particularly difficult [49,376]. Early studies assessed tendon dynamics using circulating or tissue concentrations of hydroxyproline and collagen-regulating enzymes [377,378], a method later employed to evaluate connective tissue damage following eccentric exercise [379,380,381]. The development of microdialysis techniques allowed for local sampling of these substances proximal to the tendon [382,383,384,385]. However, it was argued that microdialysis data may primarily reflect collagen synthesis at the tendon periphery and not necessarily represent changes within tendon itself [376]. More recently, stable isotope infusion combined with percutaneous tendon biopsies in humans has enabled more accurate assessments of tendon tissue morphology (e.g., collagen fibril diameter) and tendon molecular changes (i.e., protein turnover, mRNA transcription) in response to exercise [376]. Nevertheless, even biopsy-based studies report variability in collagen synthesis rates in tendons, ranging from 1% per 24 h [385] to 0.2% per 24 h [386,387].

### Take Home Message

Exercise induced muscle damage, along with skeletal muscle, can also negatively affect connective tissue, causing disturbances in basement membrane, fascia, aponeuroses and tendon. In response to repeated eccentric exercise, these connective tissue structures adapt via collagen turnover and extracellular matrix remodeling to improve stiffness, force transmission and joint stability. However, these adaptations require chronic eccentric exercise to occur, while significant methodological limitations still make it challenging to precisely quantify tendon damage and remodeling in vivo.

## 10. Nutritional Manipulation of Exercise-Induced Muscle Damage

Many professionals and recreational athletes use nutritional supplements or follow specific dietary interventions to enhance performance and speed up recovery from exercise-induced muscle damage. This has sparked growing interest in researching the impact of nutritional supplements on exercise-induced muscle damage, particularly because exercise disturbs cellular redox balance and triggers inflammatory processes [25,388,389]. Over the years, various supplements—such as proteins/amino acids [390,391,392], polyphenols [393,394], fatty acids [395] and antioxidants/vitamins [396,397,398,399,400,401,402,403]—have been studied to control or reduce the physiological and biochemical effects of muscle damage. However, results have been inconsistent, and no supplement has reliably been shown to enhance recovery after eccentric exercise-induced muscle damage [404,405].

Polyphenol-rich supplements have been proposed to enhance recovery by upregulating the antioxidant defense system, specifically through increasing nuclear translocation of Nrf2 in response to elevated phenolic metabolites [394,406]. Based on this, recent studies have explored fruit supplements rich in polyphenols to increase the expression of antioxidant genes and proteins, aiming to improve functional muscle recovery after exercise-induced muscle damage [407,408]. However, findings are mixed: while some studies report positive effects of fruit supplementation on recovery [284,379], others show no effect [409,410]. Even when muscle damage indices improve [407,408], there is no consistent evidence of enhanced performance. Additionally, concerns have been raised that antioxidant supplements may interfere with exercise adaptations [411,412,413,414].

**Figure 11:**
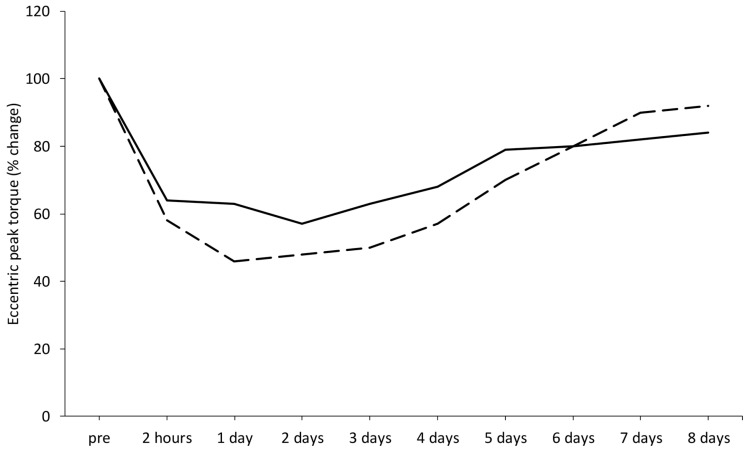
Although NAC supplementation (solid line) preserved performance recovery shortly after eccentric exercise, by the 8th day it appeared to hamper late performance recovery compared to the control group (dashed line) (adopted from [180]).

The complex effects of antioxidant supplementation were highlighted in a study using N-acetylcysteine (NAC) after eccentric muscle-damaging exercise [180] (Figure 11). While chronic NAC supplementation (over eight days) impaired strength recovery, despite initial improvements on day three, it was suggested that prolonged NAC use might inhibit key signaling pathways like p38MAPK phosphorylation and TNF-a, which are vital for muscle regeneration and protein synthesis [415,416]. These findings underscore the importance of oxidative stress in regulating muscle recovery, raising concerns about the use of supplements that could blunt inflammation or oxidative processes, potentially limiting physiological training adaptations [417].

The negative reputation of antioxidant supplements in sport nutrition and biomedicine stems from these inconsistent findings, are likely due to methodological limitations. Halliwell [418] suggested focusing on individuals with clear antioxidant deficiencies, as they may be more likely to benefit from supplementation. Following this recommendation, we used a stratified sampling approach to identify and enroll “information rich” subjects with specific characteristics [419,420,421,422]. This approach revealed that personalized antioxidant supplementation, based on specific deficiencies, can enhance exercise performance [419,420,421,422].

A key question is why athletes seek to reduce exercise-induced muscle damage using supplements, given that the antioxidant and anti-inflammatory properties of these supplements may hinder exercise adaptations. Exercise-induced muscle damage acts as a stimulus for adaptation through biochemical and inflammatory processes in response to repeated exercise [417]. The primary interest in mitigating muscle damage arises when athletes need to maintain performance and accelerate recovery between competitions, such as in tournaments or between the qualification and final races [180,406].

While various nutritional supplements, such as polyphenols, antioxidants, proteins, and fatty acids, have been studied for their potential to mitigate exercise-induced muscle damage, their effectiveness remains inconsistent. Antioxidant supplementation, in particular, presents a paradox: while it may reduce oxidative stress and inflammation, it could also blunt the very physiological processes necessary for muscle adaptation and recovery. The mixed findings in research highlight the importance of methodological rigor and individualized approaches to supplementation.

Rather than universally recommending antioxidant or anti-inflammatory supplements, a more strategic approach should be considered—targeting supplementation only for individuals with specific deficiencies or in situations where rapid recovery is essential, such as in high-frequency competition settings. Regarding exercise-induced muscle damage, indiscriminate use of supplements to minimize it may be counterproductive in the long run, since they may interfere with the natural adaptation mechanisms of the body.

### Take Home Message

Most nutritional strategies aimed at reducing exercise-induced muscle damage—especially using antioxidant and polyphenol supplementation—show inconsistent benefits and, in some cases (e.g., chronic NAC), can actually impair strength recovery by blunting the redox and inflammatory signaling that drives adaptation. Rather than routinely trying to control exercise induced muscle damage, supplements should be used for situations with documented deficiencies or for periods of repeated competition when the recovery window is very short. In typical training contexts, indiscriminate antioxidant or anti-inflammatory use may be counterproductive, interfering with the cellular stress responses that underpin long-term performance.

## 11. Future Research and Advanced Methods in Eccentric Exercise

Future studies on eccentric exercise-induced muscle damage and adaptation would benefit from the implementation of advanced methodological approaches. Omics techniques, including transcriptomics, proteomics and metabolomics, offer the potential to capture global molecular signatures associated with different eccentric loading paradigms and recovery profiles, helping to identify pathways that may not be evident from traditional approaches alone. Complementary single-fiber analyses can provide high-resolution information on fiber type-specific responses, sarcomeric disorganization, excitation–contraction coupling disturbances and mitochondrial alterations that are masked in whole muscle samples. In addition, the integration of these methods with other state-of-the-art techniques (e.g., high-content imaging, multiplex assays, and refined in vivo functional testing) may allow a more precise description of structural and functional damage versus adaptive remodeling. To understand this potential, appropriately powered studies with carefully justified sample sizes are essential [423], as underpowered designs limit the interpretability and reproducibility of complex datasets. Together, these methodological advances are likely to provide a more comprehensive and mechanistically robust understanding of how eccentric exercise perturbs, and ultimately reshapes, skeletal muscle.

## 12. Conclusions

In this review, we have highlighted how eccentric exercise, while often associated with exercise-induced muscle damage, is also a stimulus for structural, neural, and metabolic adaptation. Importantly, exercise-induced muscle damage should not be viewed as a uniform outcome of all eccentric exercise. Rather, its magnitude, time course, and mechanistic profile depend on the eccentric methodology applied, since different eccentric exercise models impose distinct loading characteristics, muscle lengths, contraction velocities, neuromuscular demands, and degrees of familiarity with the stimulus. The relevant sections of the review are summarized in the following numbered points, which present the current understanding of the mechanisms underpinning eccentric exercise-induced muscle damage, the subsequent repair and remodeling processes, and their implications for performance and long-term muscle health.

1. Eccentric exercise-induced muscle damage is mainly the result of unaccustomed high mechanical tension during lengthening contractions, which is further influenced by long muscle length and high contraction velocity. Importantly, the extent of muscle damage also depends on the eccentric methodology employed, as different exercise models impose different mechanical and physiological demands. Indirect biomarkers of exercise induced muscle damage do not describe the true extent of structural myofiber damage but instead capture changes in distinct physiological processes.
2. Following eccentric exercise, skeletal muscle repair is achieved through a coordinated response in which satellite cells are key, but not always indispensable, players, working alongside inflammatory, and intrinsic membrane–repair mechanisms. The severity of exercise induced muscle damage and the nature of the exercise performed influence the extent to which regeneration depends on satellite cells. However, the role of satellite cells in long-term adaptations and in responses to non-damaging exercise remains unresolved.
3. Eccentric exercise uniquely combines high force output performed with low energy demands. At the same time, resting energy expenditure remains elevated for several days after eccentric exercise due to muscle repair processes, protein synthesis and increased fat oxidation. These characteristics make eccentric exercise a potent tool for both performance enhancement and metabolic conditioning.
4. After an initial bout of damaging eccentric exercise session, subsequent sessions of the same nature progressively cause less muscle damage, a phenomenon known as the repeated bout effect. This adaptive “memory” of skeletal muscle involves neural, architectural, connective tissue and molecular adaptations. Over time, eccentric training becomes no more injurious than other modes of resistance training and, when strategically alternated and combined with concentric work, may optimally enhance muscle function, resilience and health, particularly in populations such as older adults.
5. Eccentric training shares distinct, often region-specific architectural adaptations, most notably increased fascicle length and altered morphology achieved via a mix of sarcomere- and tendon-level changes. Interestingly, eccentric training shares similar hypertrophic signaling with concentric training, so its efficient and high-force benefits should be leveraged within carefully designed programs.
6. Eccentric exercise is controlled by the nervous system in a fundamentally different way than concentric exercise characterized by lower neural activation for the same or greater force, enhanced cortical drive with concurrent spinal inhibition, and a planned down-weighting of spindle input. Acute eccentric exercise may cause impairments in neuromuscular performance and proprioception, yet chronic eccentric exercise induces neural adaptations, including altered motor unit recruitment, improved proprioception and cross-education effects, that in turn enhance control, protection, and strength over time.
7. Acute eccentric exercise disrupts mitochondrial and sarcoplasmic reticulum structure, by elevating calcium and reactive oxygen species levels, thereby triggering mitophagy. However, during repeated bouts of eccentric exercise, these same stress signals drive targeted remodeling of mitochondria, the sarcoplasmic reticulum and the cytoskeleton, restoring function, enhancing excitation–contraction coupling, and improving the muscle’s resilience to future mechanical loads.
8. Acute eccentric exercise causes perturbations in connective tissue, namely fascia, tendon and extracellular matrix, which likely contribute to the development of DOMS. With chronic eccentric exercise, connective tissue adapts via collagen turnover and structural remodeling, enhancing stiffness, force transmission and joint stability. However, the precise role of these adaptations in exercise-induced muscle damage and recovery remains difficult to define, as they occur slowly and are challenging to capture with available research tools.
9. Nutritional strategies to limit exercise-induced muscle damage, especially antioxidant and polyphenol supplementation, have shown inconsistent benefits and may even blunt training adaptations. These supplements should therefore be used sparingly, targeted to true deficiencies or tight competition schedules rather than applied routinely.

Overall, eccentric exercise should not be regarded as inherently damaging, but rather as a potent and highly context-dependent mechanical stimulus whose acute damaging effects and long-term adaptations are shaped by the specific loading conditions under which it is performed. A clearer distinction between eccentric methodologies may therefore help explain the variability in findings across studies and improve the design of future mechanistic, applied and clinical research.

## Data Availability

No new data were created or analyzed in this study. Data sharing is not applicable to this article.
